# Effectiveness and safety of ustekinumab in pediatric Crohn's disease: Results of the REALITI study

**DOI:** 10.1002/jpn3.70372

**Published:** 2026-03-02

**Authors:** Steven J. Steiner, Jeremy Adler, Shehzad A. Saeed, Richard S. Strauss, Kristin M. Howe, Anna Sheahan, Renping Zhang, Kimberly R. Keihn, Katharine Harrow, Kelly K. Olano, David A. Evans, Kim Hung Lo, Nanhua Zhang, Lilianne Kim, Richard B. Colletti, Sheri Volger, for the ImproveCareNow Learning Health Network, Sabina Ali, Rana Ammoury, Keren L. Appel, Travis Ayers, Howard Baron, Julie Bass, Rachel Bensen, Erica J. Brenner, José Cabrera, Mallory Chavannes, J Fernando del Rosario, Jill Dorsey, Dana M. H. Dykes, Dawn R. Ebach, Bhaskar Gurram, Dyer Heintz, Leslie M. Higuchi, Edward J. Hoffenberg, Jeannie S. Huang, Esther Jacobowitz Israel, Traci W. Jester, Ian Kang, Ian Leibowitz, Tiffany Linville, Ross M. Maltz, Craig A. McKinney, Lybil B. Mendoza Alvarez, Phillip Minar, Tina Morhardt, Alisa J Muñiz Crim, Barbara Joanna Niklinska‐Schirtz, Daniel M. O'Connell, Helen Pappa, Brad Pasternak, Trusha Patel, Meryl K. Perlman, Kelly C. Sandberg, Marc E. Schaefer, Robbyn E. Sockolow, David L. Suskind, Gitit Tomer, Jeanne Tung, Amanda A. Wenzel, Denise D. Young

**Affiliations:** ^1^ Riley Hospital for Children Indiana University School of Medicine Indianapolis Indiana USA; ^2^ C.S. Mott Children's Hospital, Michigan Medicine University of Michigan Ann Arbor Michigan USA; ^3^ Dayton Children's Hospital/Wright State University Boonshoft School of Medicine Dayton Ohio USA; ^4^ Johnson & Johnson Spring House Pennsylvania USA; ^5^ ImproveCareNow Essex Junction Vermont USA; ^6^ Johnson & Johnson Horsham Pennsylvania USA; ^7^ Johnson & Johnson La Jolla California USA; ^8^ Cincinnati Children's Hospital Medical Center Cincinnati Ohio USA; ^9^ Research Data Corporation Gibbsboro New Jersey USA; ^10^ University of Cincinnati College of Medicine Cincinnati Ohio USA; ^11^ University of Vermont Children's Hospital Burlington Vermont USA

**Keywords:** biologic product, children, inflammatory bowel disease, real‐world, registries

## Abstract

**Objectives:**

Few approved treatments exist for children with Crohn's disease (CD). The REALITI study retrospectively assessed the effectiveness and safety of ustekinumab in real‐world clinical settings for children with CD.

**Methods:**

Data were collected from the prospective *ImproveCareNow* (ICN) registry for pediatric patients (≥ 2 to <18 years old) and young adult patients (≥ 18 to <26 years old), regardless of baseline CD severity. Additional analyses were conducted for a subset of patients who had moderately‐to‐severely active CD (short pediatric CD activity index [sPCDAI] ≥30). Key Week‐52 endpoints included clinical remission (sPCDAI ≤10) and corticosteroid‐free (CF) clinical remission. Safety events of interest were assessed at Week 52.

**Results:**

Overall, 479 patients with CD were treated with ustekinumab, 348 pediatric patients and 131 young adults; most were biologic‐exposed (pediatric, 98.9%; young adult, 95.4%). At Week 52 (observed case; excluding patients without Week 52 data), clinical remission was achieved by 47.3% (125/264) of pediatric patients and 44.8% (39/87) of young adults, and CF clinical remission by 41.3% (109/264) and 39.1% (34/87), respectively. At Week 52 (observed case), among patients with moderately‐to‐severely active CD, clinical remission was achieved by 36.9% (41/111) of pediatric patients and 34.3% (12/35) of young adults, and CF clinical remission by 31.5% (35/111) and 28.6% (10/35), respectively. Ustekinumab was well tolerated, with no new safety signals identified.

**Conclusions:**

In the REALITI study of real‐world data from the ICN registry, the effectiveness and safety of ustekinumab treatment through 52 weeks were similar in pediatric and young adult patients with CD.

**Trial Registration:**

ClinicalTrials.gov identifier: NCT05242458; https://clinicaltrials.gov/study/NCT05242458

## Abstract (highlights)

**What is Known:**

- Ustekinumab is efficacious and safe in adults with moderately‐to‐severely active Crohn's disease (CD).
- Ustekinumab has yet to be approved globally for pediatric patients with CD.

**What is New:**

- The retrospective real‐world evidence REALITI study used data from the *ImproveCareNow* registry and found that the effectiveness and safety of ustekinumab treatment through 52 weeks were similar among pediatric patients and a reference population of young adult patients.
- This is a novel demonstration of the value of a large pediatric inflammatory bowel disease registry to provide supportive evidence for the regulatory approval of a CD treatment, with the potential for improving treatment access and outcomes in pediatric CD.

## 1 INTRODUCTION

Despite the increasing prevalence of pediatric inflammatory bowel disease (IBD) in recent decades, few approved treatment options exist for pediatric patients.^1^, ^2^, ^3^, ^4^, ^5^ Delays in regulatory approvals of Crohn's disease (CD) therapies for pediatric patients often lead to off‐label use, raising concerns about potential for incorrect dosing and unknown risk to pediatric patients.^6^ Real‐world data (RWD) sources, such as patient disease registries, provide valuable data on a wide range of outcomes in large populations and can be used to support the evaluation of the effectiveness of CD therapies in pediatric patients.

Ustekinumab, an interleukin‐12/23 p40 subunit antagonist, has been shown to be an effective and safe therapy in adults with moderately‐to‐severely active CD and was approved by the U.S. Food and Drug Administration (FDA) and the European Medicines Agency (EMA) for the treatment of adult patients in 2016.^7^, ^8^ Recently, ustekinumab was approved for use in pediatric patients with CD in the European Union, specifically for those weighing ≥40 kg.^9^ Despite lack of global regulatory approvals, ustekinumab has been used off‐label in the pediatric CD population.^10^, ^11^

*ImproveCareNow* (ICN) is a collaborative pediatric IBD quality improvement and research network consisting of over 100 pediatric gastroenterology care centers. The registry currently houses prospectively collected point‐of‐care data from more than 50,000 patients with CD or ulcerative colitis, representing a large proportion of the pediatric IBD patients in the United States.^12^ The registry allows researchers to perform retrospective studies, thus informing the care of pediatric patients with IBD.^13^, ^14^, ^15^, ^16^, ^17^, ^18^ The REALITI study is a real‐world evidence (RWE) study that used data from the ICN registry to assess the effectiveness and safety of ustekinumab for CD in pediatric patients ≥2 to <18 years old in comparison to young adult patients ≥18 to 26 years old who were treated with ustekinumab for 1 year in routine clinical practice. In addition, a subset of patients with moderately‐to‐severely active CD was studied to support applications for regulatory approval. Here, we present a broad scope of the effectiveness and safety of ustekinumab in pediatric patients with CD in a real‐world setting from pediatric patients ≥2 to <18 years old in the ICN registry, regardless of weight.

## 2 METHODS

### 2.1 Ethics statement

Institutional Review Board (IRB) approval was obtained either at each site participating in the ICN registry or by the central IRB (Cincinnati Children's Hospital Institutional Review Board), as applicable.

### 2.2 Study design and participants

The REALITI study (ClinicalTrials.gov identifier: NCT05242458) is a retrospective, non‐interventional, observational, RWE cohort study designed to evaluate RWD from the ICN registry on the effectiveness and safety of ustekinumab treatment in pediatric patients. Because this study did not have a concurrent active control group, young adult patients ≥18 to <26 years old were identified as the most appropriate reference group. The primary analysis was conducted on all patients who started ustekinumab regardless of clinical disease activity. An additional analysis was conducted for a subset of pediatric patients with moderately‐to‐severely active CD compared to a subset of young adult patients with moderately‐to‐severely active CD.

The ICN registry includes detailed demographic characteristics and relevant CD‐related clinical characteristics (phenotype and extent of disease [Paris classification]^19^). Also collected are serial measures of CD activity (short Pediatric CD Activity Index [sPCDAI]) data components^20^), laboratory assessments, and safety outcomes, including serious infections and IBD‐related surgeries and hospitalizations.

For this study, additional clinical data were extracted (retrospectively) from patient medical records by trained personnel using ICN standardized data collection procedures and a study‐specific case report form maintained in Hive Networks, Inc. and were merged with the existing ICN registry data for analysis. The data collection period spanned from January 2010 through February 2020.

Eligible patients had a documented diagnosis of CD and were ≥2 to <26 years old at ustekinumab initiation (on or before June 22, 2019; intravenous or subcutaneous route; regimen determined by their physician), had ≥1 ICN visit with documented new use of ustekinumab, had ≥1 ICN visit before ustekinumab initiation and ≥1 record in the ICN registry database after ustekinumab initiation but before February 29, 2020, and they or their legal guardian provided informed consent for the use of their ICN data for research purposes. Patients who used ustekinumab before ICN registry enrollment were excluded. Moderately‐to‐severely active CD was defined as sPCDAI ≥30 at baseline.

### 2.3 Study endpoints

The primary endpoint was clinical remission, defined as sPCDAI ≤10 at Week 52, without intercurrent events (ICEs) before Week 52. Patients who had any of the following ICEs were considered not to be in clinical remission: (1) discontinuation of ustekinumab due to worsening of CD or due to lack of effectiveness as assessed by medical record review; or (2) initiation of cyclosporine, tacrolimus, or biologic agents before the date of the Week 52 endpoint assessment or the ustekinumab discontinuation date. The sPCDAI is a six‐component shorter version of the Pediatric CD Activity Index (PCDAI) that does not require laboratory scores, height measurements, or a perianal exam score.^20^ Results from the sPCDAI are positively correlated with the full PCDAI and the Physician's Global Assessment in pediatric and young adult patients.^20^ The sPCDAI cumulative score ranges from 0 to 90, with higher scores indicating more severe disease activity. Week 52 was calculated as the date of the first dose of ustekinumab (index date) plus 365 days. The Week‐52 endpoint value was defined as the measurement closest to Week 52, in a window defined as Week 52 ± 16 weeks.

Secondary clinical effectiveness endpoints included corticosteroid‐free clinical remission (sPCDAI ≤10 without use of corticosteroids at Week 52); clinical response (a reduction of ≥10 points in the sPCDAI from baseline to Week 52) among patients with moderately‐to‐severely active CD; and changes from baseline at Week 52 in growth parameters, including height, weight, and body mass index (BMI) *z* scores. Additional endpoints included the rate of ustekinumab discontinuation, time to ustekinumab discontinuation, and reasons for ustekinumab discontinuation (collected retrospectively).

Safety events of interest were frequency of (1) IBD‐related hospitalizations and surgeries (2) and adverse events of special interest, which included serious infections, opportunistic infections, tuberculosis, malignancy, anaphylaxis requiring ustekinumab discontinuation, and death. Change from baseline at Week 52 in hematocrit (HCT), albumin, and erythrocyte sedimentation rate (ESR) was also determined.

### 2.4 Statistical analysis

All data were summarized descriptively; no hypothesis testing and no comparative statistical analyses were performed. Descriptive statistics, such as mean, median, standard deviation, and interquartile range, were used to summarize continuous variables. Counts and percentages were used to summarize categorical variables. Graphs were also used to summarize data.

Demographics and baseline characteristics were based on the measurement closest to the index date within the baseline window (12 weeks before to 2 weeks after ustekinumab initiation), except for disease and medication history, for which data from all visits before or on the index date were used.

Both observed case and intention‐to‐treat analyses were conducted in both the primary cohort and in the subset of patients with moderately‐to‐severely active CD. First, we conducted an observed case analysis in which patients without an available clinical remission status in the Week‐52 window were excluded from the analysis. In this analysis, the ICE criteria were applied. Second, we conducted an intention‐to‐treat analysis in which patients who had missing clinical remission status at Week 52, after accounting for ICEs, were considered not to be in clinical remission. Subgroup analyses (observed case) were conducted for clinical remission at Week 52 among all pediatric and young adult patients who increased the dose or frequency of ustekinumab from their initial maintenance dosing regimen by Week 52.

Clinical remission status was determined algebraically using the complete or partial sPCDAI as follows: if the complete sPCDAI score was ≤10, then clinical remission was determined as achieved; or, if the complete or partial sPCDAI score was >10, then the patient was not in clinical remission; or, if the partial sPCDAI score was equal to zero with only one missing component (maximum score of 10), then clinical remission was determined as achieved.

Survival curves for time to ustekinumab discontinuation were estimated using the Kaplan–Meier method. Patients who did not discontinue ustekinumab were censored at the date of the last dose of ustekinumab or the end date of the Week‐52 window (i.e., Week 52 plus 16 weeks), whichever came first.

Safety events of interest were collected in the case report form during the medical record review. The proportion of patients who experienced ≥1 occurrence of a given safety event between initial ustekinumab exposure and the earlier date of Week 52 or February 29, 2020 were summarized.

## 3 RESULTS

A total of 479 patients ≥2 to <26 years old with CD were identified in the ICN registry as having initiated ustekinumab and met all inclusion criteria within the time range of the study, 348 of whom were pediatric patients and 131 were young adult patients. A majority of patients in each cohort were White (pediatric, 77.9%; young adult, 82.4%; Table 1). Most (89.4%) of the pediatric patients were 12 to <18 years old. The pediatric and young adult cohorts had similar baseline demographics and CD characteristics except, as expected, age, median weight, median disease duration, and proportions of patients who had stricturing CD phenotype, or had prior CD‐related surgeries. Severe disease (sPCDAI >40) was observed for 25.3% of pediatric patients and 24.1% of young adult patients. Nearly all patients were biologic‐exposed (pediatric, 98.9%; young adult, 95.4%), with 59.8% of pediatric patients and 58.0% of young adults having prior exposure to two or more unique non‐simultaneous biologics. The proportions of patients who were receiving corticosteroids at ustekinumab initiation was 47.1% for pediatric patients and 50.4% for young adults.

**Table 1:** Baseline demographics, disease characteristics, and medication and biologic exposure history.

	Pediatric patients	Young adult patients
	*N* = 348	*N* = 131
Demographics		
Age, yrs, median (IQR)	15.0 (13.0; 16.0)	19.0 (18.0; 20.0)
Age range, yrs	4; 17	18; 24
Age group, yrs, *n* (%)		
2 to 6	3 (0.9)	—
6 to <12	34 (9.8)	—
12 to <18	311 (89.4)	—
18 to <26	—	131 (100)
Female, *n* (%)	179 (51.4)	74 (56.5)
Race, *n* (%)		
White	271 (77.9)	108 (82.4)
Black or African American	31 (8.9)	10 (7.6)
Unknown	23 (6.6)	7 (5.3)
Other	16 (4.6)	6 (4.6)
Asian	6 (1.7)	—
American Indian or Alaska Native	1 (0.3)	—
Weight, kg	*N* = 344	*N* = 129
Median (IQR)	50.10 (40.45; 60.15)	63.05 (54.34; 73.57)
<40	81 (23.5)	2 (1.6)
≥40	263 (76.5)	127 (98.4)
Weight *z* score median (IQR)	−0.30 (−1.34; 0.49) *N* = 290	0.09 (−0.97; 0.86) *N* = 83
Height *z* score, median (IQR)	−0.43 (−1.17; 0.36) *N* = 289	−0.33 (−0.88; 0.45) *N* = 83
BMI *z* score, median (IQR)	−0.25 (−1.26; 0.68) *N* = 289	−0.21 (−0.83; 0.98) *N* = 83
Minimum BMI *z* score since CD diagnosis prior to index date, median (IQR)	−0.77 (−1.82; 0.09)	−0.37 (−1.61; 0.52)
Disease characteristics		
Age at diagnosis, yrs, median (IQR)	10.0 (7.5; 12.0)	13.0 (10.0; 16.0)
CD duration, yrs, median (IQR)	3.93 (2.23; 6.16) *N* = 327	6.14 (3.43; 8.86) *N* = 120
Involved lower GI tract areas,^a^ *n* (%)	*N* = 343	*N* = 130
Ileum only	39 (11.4)	14 (10.8)
Colon only	62 (18.1)	13 (10.0)
Ileum and colon	238 (69.4)	103 (79.2)
None	4 (1.2)	—
CD phenotype,^a^ *n* (%)	*N* = 344	*N* = 130
Inflammatory/non‐penetrating/non‐stricturing	256 (74.4)	93 (71.5)
Stricturing only	40 (11.6)	23 (17.7)
Penetrating only	25 (7.3)	5 (3.8)
Both stricturing and penetrating	2 (0.6)	—
Unknown	21 (6.1)	9 (6.9)
Prior CD‐related surgeries,^a^ *n* (%)	36 (10.3)	22 (16.8)
sPCDAI score at baseline, median (IQR)	25.0 (10.0; 45.0) *N* = 292	22.5 (10.0; 40.0) *N* = 116
≤10 (inactive), *n* (%)	80 (27.4)	39 (33.6)
>10 to <30 (mild), *n* (%)	67 (22.9)	25 (21.6)
≥30 (moderate to severe), *n* (%)	145 (49.7)	52 (44.8)
>40 (severe), *n* (%)	74 (25.3)	28 (24.1)
Max sPCDAI score since diagnosis prior to index date, median (IQR)	45.0 (30.0; 55.0) *N* = 344	40.0 (25.0; 50.0) *N* = 129
>40 (severe), *n* (%)	174 (50.6)	55 (42.6)
Time from maximum sPCDAI to index date (weeks), median (IQR)	−41.9 (−116.3; −9.9)	−46.7 (−136.4; −10.0)
Prior biologic exposure,^a^ *n* (%)		
Infliximab	291 (83.6)	107 (81.7)
Adalimumab	231 (66.4)	87 (66.4)
Golimumab	5 (1.4)	1 (0.8)
Certolizumab	25 (7.2)	20 (15.3)
Natalizumab	1 (0.3)	1 (0.8)
Vedolizumab	81 (23.3)	26 (19.8)
Number of prior unique biologics,^a^ *n* (%)		
None	4 (1.1)	6 (4.6)
1 biologic	136 (39.1)	49 (37.4)
2 biologics	141 (40.5)	43 (32.8)
≥3 biologics	67 (19.3)	33 (25.2)
Baseline^b^ disease‐related medications, *n* (%)		
Thiopurines	35 (10.1)	11 (8.4)
Methotrexate	98 (28.2)	26 (19.8)
Corticosteroids	164 (47.1)	66 (50.4)

*Note*: Baseline value is defined as the measurement closest to the index date within the baseline window (i.e., from −12 weeks to +2 weeks from the index date); the index date is defined as the date of the first dose of ustekinumab; IQR is defined as the range between the 25th and 75th percentiles of the data.

Abbreviations: BMI, body mass index; CD, Crohn's disease; GI, gastrointestinal; IQR, interquartile range; sPCDAI, short Pediatric CD Activity Index; yrs, years.

^a^ Assessed using data from all visits prior to or on the index date.

^b^ During the baseline window (i.e., from −12 weeks to +2 weeks from the index date).

### 3.1 Exposure

The route of the first induction dose was IV for 94.3% of the pediatric patients and 94.7% of the young adult patients. Approximately 93% of patients in each cohort received an initial ustekinumab induction dose of 260–520 mg (approved adult CD dose, equivalent to an IV dose of approximately 0.5 to <1.65 mg/kg; Table S1). A majority of patients in each cohort received an initial maintenance dose of 90 mg every 8 weeks (q8w; pediatric patients, 83.0%; young adults, 88.3%). By Week 52, the proportion of pediatric patients receiving 90 mg q8w had decreased from 83.0% to 52.2%, whereas the proportion of pediatric patients receiving 90 mg q4w increased from 7.2% to 34.4%. By Week 52, the proportion of young adult patients receiving 90 mg q8w had decreased from 88.3% to 64.7%, whereas the proportion of young adult patients receiving 90 mg q4w increased from 4.2% at initial maintenance to 21.8% at final maintenance.

### 3.2 Clinical effectiveness

**Figure 1:**
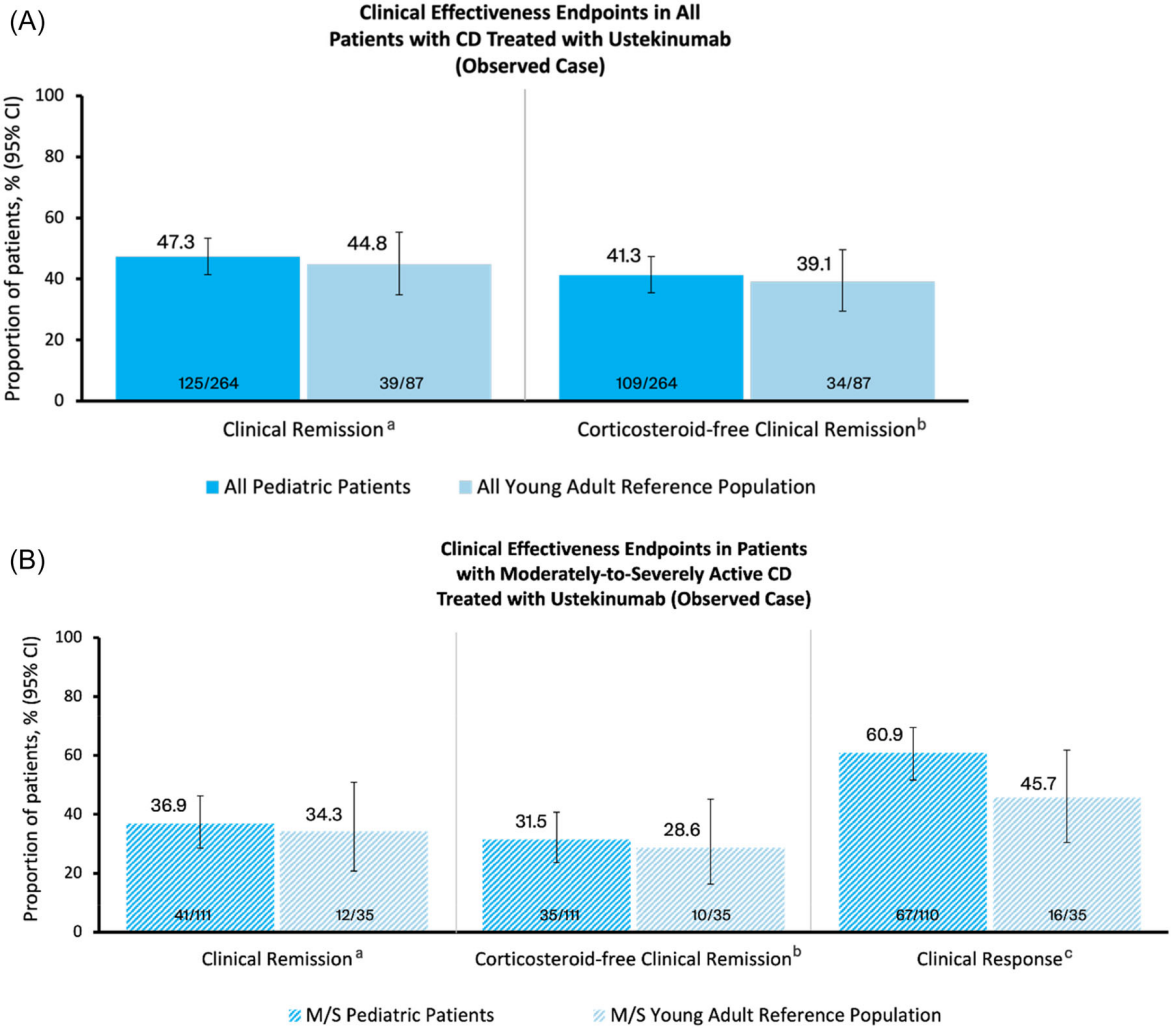
Observed case analysis of clinical effectiveness endpoints at Week 52 in (A) all patients with CD treated with ustekinumab, and (B) patients with moderately‐to‐severely active CD treated with ustekinumab. *Note*: For this observed case analysis, patients with missing clinical remission status at Week 52 after accounting for ICEs were excluded. The Week‐52 window was defined as Week 52 ± 16 weeks. Week 52 was calculated as the date of the first dose of ustekinumab plus 365 days. The 95% CI was estimated based on the Wilson method. CD, Crohn's disease; CI, confidence interval; ICE, intercurrent event; M/S, moderately‐to‐severely active CD (sPCDAI ≥30 at baseline); sPCDAI, short Pediatric Crohn's disease Activity Index. ^a^Clinical remission was defined as sPCDAI ≤10 at Week 52. ^b^Corticosteroid‐free clinical remission was defined as sPCDAI ≤10 without use of corticosteroids at Week 52. ^c^Clinical response was defined as a reduction of ≥10 points in the sPCDAI from baseline to Week 52.

Of the 348 patients ≥2 to <18 years old, 264 had a clinical remission assessment in the Week‐52 window, and were included in the observed case analysis. Of the 131 patients ≥18 to 26 years old, 87 had a clinical remission assessment in the Week‐52 window, and were included in the observed case analysis. In the observed case analysis at Week 52, similar proportions of pediatric patients and young adult patients achieved clinical remission (pediatric, 47.3% [125/264]; young adult, 44.8% [39/87]) and corticosteroid‐free clinical remission (pediatric, 41.3% [109/264]; young adult, 39.1% [34/87]) (Figure 1A). Among the subset of patients with moderately‐to‐severely active CD, similar proportions of pediatric patients and young adult patients achieved clinical remission (pediatric, 36.9% [41/111]; young adult, 34.3% [12/35]) and corticosteroid‐free clinical remission (pediatric, 31.5% [35/111]; young adult, 28.6% [10/35]) (Figure 1B). In addition, for the patients with moderately‐to‐severely active CD, clinical response was achieved by 60.9% (67/110) of pediatric patients and 45.7% (16/35) of young adult patients (Figure 1B).

In the intention‐to‐treat analysis at Week 52, for all patients, clinical remission was achieved by 35.9% (125/348) of pediatric patients and 29.8% (39/131) of young adults, and corticosteroid‐free clinical remission by 31.3% (109/348) and 26.0% (34/131), respectively (Figure S1a). Among the subset of patients with moderately‐to‐severely active CD, clinical remission was achieved by 28.3% (41/145) of pediatric patients and 23.5% (12/51) of young adults, and corticosteroid‐free clinical remission by 24.1% (35/145) and 19.6% (10/51), respectively (Figure S1b). Among all patients who increased ustekinumab dose or frequency and had a clinical remission assessment in the Week‐52 window (observed case), clinical remission at Week 52 was achieved by 40.0% (42/105) of pediatric patients and 37.5% (12/32) of young adults (Figure S2).

A positive median change from baseline in weight and BMI *z* score (for age and sex) was observed for both cohorts, but there was no evidence of improvement in height *z* score or catch‐up growth (Table S2). From baseline to Week 52, for the pediatric patients and the young adult patients, respectively, HCT increased by 1.1% and 1.2%, albumin increased by 2.0 and 3.0 g/L, and ESR decreased by −5.0 and −4.5 mm/h (Table S3).

**Figure 2:**
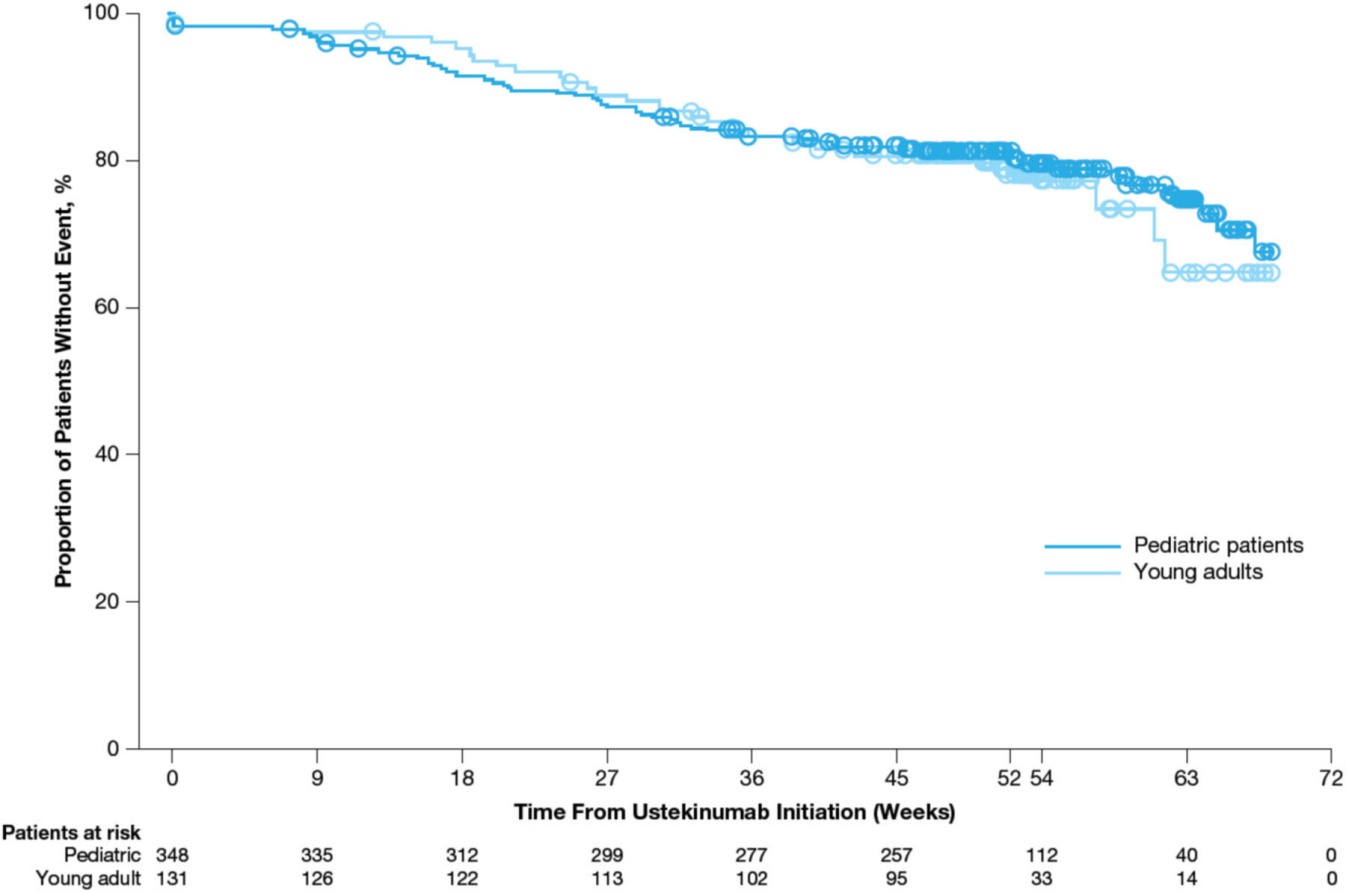
Kaplan–Meier plot of time to discontinuation of ustekinumab. *Note*: Patients who discontinued ustekinumab were considered to have had an event, and the date of their ustekinumab discontinuation was used in the time to event calculation. Patients who did not have an event (i.e., ustekinumab discontinuation) were censored at the date of the last dose of ustekinumab during the study period or the end date of the Week‐52 window (i.e., Week 52 plus 16 weeks), whichever came first.

The Kaplan–Meier survival rate of ustekinumab discontinuation over time was similar in pediatric patients and young adult patients (Figure 2). The proportion of patients who discontinued ustekinumab through Week 52 was low and similar for pediatric patients (21.0%) and young adult patients (22.9%), with discontinuations due to intolerance or a serious adverse event <1.0% for both cohorts (Table S4).

### 3.3 Safety events of interest

Overall, 31.9% of pediatric patients and 19.8% of young adult patients had IBD‐related hospitalizations, with 16.1% and 9.2%, respectively, having IBD‐related surgeries (Table 2). The most common adverse event of special interest was serious infections, which occurred in a larger proportion of pediatric patients (7.5%) than young adult patients (3.8%). Rates of opportunistic infections were low in both pediatric patients (1.4%) and young adult patients (0%). No tuberculosis, malignancy, or anaphylaxis requiring ustekinumab discontinuation occurred in either cohort through Week 52. One death (cardiopulmonary arrest) was reported in the pediatric patient cohort. The death was deemed by investigators to be unrelated to IBD or ustekinumab treatment.

**Table 2:** Safety events of interest through Week 52.

	Pediatric patients, *N* = 348	Young adult patients, *N* = 131
Patients with ≥1 safety event of interest,^a^ *n* (%)	120 (34.5)	28 (21.4)
IBD‐related hospitalization	111 (31.9)	26 (19.8)
IBD‐related surgery	56 (16.1)	12 (9.2)
Adverse events of special interest		
Serious infection	26 (7.5)^b^	5 (3.8)^c^
Opportunistic infections	5 (1.4)^d^	0
Tuberculosis	0	0
Malignancy	0	0
Anaphylaxis requiring discontinuation of ustekinumab	0	0
Death	1 (0.3)^e^	0

Abbreviation: IBD, inflammatory bowel disease.

^a^ Patients were counted only once for any given event, regardless of the number of times they experienced the event.

^b^ Two cases each of *Clostridium difficile* and *Staphylococcus* bacteremia, and one case each of pharyngitis, viral infection, breast abscess, pyosalpinx, and recurrence of phlegmon; along with two cases of perianal abscess, an expected disease outcome.

^c^ One case each of perianal infection and *Staphylococcus aureus*.

^d^ One case each of *Staphylococcus epidermidis* bacteremia and *Cryptosporidium*.

^e^ The death (cardiopulmonary arrest) was deemed by investigators as unrelated to IBD or ustekinumab treatment.

## 4 DISCUSSION

The REALITI study was a retrospective, observational, RWE study designed to analyze RWD from the ICN registry to assess the effectiveness and safety of ustekinumab in pediatric patients with CD. The reference group consisted of young adult patients with CD, for whom ustekinumab has been approved by the FDA and the EMA. The key finding was that similar proportions of pediatric and young adult patients receiving ustekinumab induction and maintenance therapy over 52 weeks achieved clinical remission. Clinical remission was also achieved by similar proportions of pediatric and young adult patients with moderately‐to‐severely active CD without an increase in adverse events. Even though a larger proportion of pediatric patients than young adult patients dose escalated, clinical remission was maintained in comparable proportions in both cohorts. Information regarding the reasons for dose escalation was not collected, but dose escalation is common with biologic treatment of pediatric CD in patients who have incomplete response to standard dosing, including failure to achieve remission or secondary loss of response.^21^ The proportions of patients discontinuing ustekinumab by Week 52 were low in both cohorts, suggesting a sustained treatment benefit. Additionally, trends toward improvements in weight gain and key laboratory parameters were observed in the pediatric patients. Finally, limited safety data were collected by the registry, and thus, these data should be interpreted cautiously. Nevertheless, the safety findings along with low rates of ustekinumab discontinuation suggest that ustekinumab is generally well tolerated for the treatment of pediatric CD in routine clinical practice.

Two retrospective real‐world cohort studies of pediatric patients with CD treated with ustekinumab (both, *N* = 101) reported 1‐year remission rates of 80% (sPCDAI <15 without concomitant steroids in the previous 30 days) and 40.5% (corticosteroid‐free remission or exclusive enteral nutrition‐free remission [weighted PCDAI < 12.5]).^11^, ^22^ In comparison, the 1‑year remission rate for all pediatric patients in the current REALITI study was 47.3% (sPCDAI ≤10) and the corticosteroid‐free remission rate was 41.3% (sPCDAI ≤10 without use of corticosteroids at Week 52). In the previous studies, the definitions of remission were less stringent than in the current study, and the statistical analysis did not use the same ICE criteria as the current study, making direct comparisons challenging.

Patients in this study represented a more clinically and racially diverse population compared with clinical trials and previously published studies, offering a more comprehensive representation of pediatric patients with CD in real‐world settings. Unlike the strict inclusion and exclusion criteria and prolonged screening processes employed in randomized clinical trials (RCTs), this RWE study used data from centers that seek to enroll all of their pediatric and young adult patients with CD, not just a sample, in the ICN registry. As a result, this study provides valuable insights into the impact of ustekinumab on the real‐world pediatric CD population.

The majority of the patients were treatment‐refractory, with many receiving corticosteroids or immunomodulators at the time of ustekinumab initiation, and most having been exposed to other biologic therapy. The overall disease history of the pediatric patients, including prior CD‐related medication history, concomitant therapies (potential lack of wash‐out periods), and surgical history, likely rendered a large proportion of the REALITI study population ineligible for enrollment in the ongoing Phase 3 UNITI Jr. clinical trial (ClinicalTrials.gov identifier: NCT04673357). However, these exclusions make this study population more reflective of patients cared for in real‐world practice. This observation also aligns with a study showing that a significant proportion of children with IBD who initiated biologics in real‐world settings would not meet the stringent inclusion criteria of RCTs.^23^ Additionally, in that study, patients ineligible for RCT participation often had worse clinical outcomes than those who met eligibility criteria.

To facilitate a comparison of the data from this study with those from RCTs, inclusion and exclusion criteria were established and aligned with the typical RCT criteria (additional analyses of patients with moderately‐to‐severely active CD). However, as with any retrospective observational database study, it is important to acknowledge the associated limitations when interpreting the results of the REALITI study. First, the lack of an active, concurrent control group led to young adult patients treated within ICN being selected as the most appropriate active reference group; nonetheless, sPCDAI scores are used as a standard part of practice at each ICN clinic visit, and the young adult reference population was treated by the same care teams as the pediatric population. Second, the lack of randomization makes the analysis more likely to be impacted by unobserved confounding factors. Third, a majority (89.4%) of the pediatric patients were 12–17 years old and most (76.5%) weighed ≥40 kg. Thus, further evaluations of ustekinumab in younger pediatric patients with CD and in those weighing <40 kg are still needed. Further, the 1‐year follow‐up period prevented capturing longer‐term real‐world outcomes. Finally, although safety data were collected prospectively during routine clinical practice, completeness of the safety data was limited because ICN does not have predefined safety data collection requirements for reporting adverse events or any assessments of adverse event severity, relatedness, or timing in relation to dosing, comorbidities, or concomitant medications. Detailed information on hospitalizations and surgeries was also not available. The medical record review provided some opportunity for data clarification; however, this too was limited to the information reported by the clinical teams. These are typical challenges that retrospective and real world studies often encounter when aiming to capture consistent and complete data.

## 5 CONCLUSION

In conclusion, this analysis of RWD from the ICN registry demonstrated that similar proportions of pediatric and young adult patients with CD treated with ustekinumab achieved clinical remission. Ustekinumab was well tolerated in pediatric patients, with no new safety signals identified. The REALITI study provides valuable RWE of the similar effectiveness of ustekinumab in both pediatric and young adult patients with CD in routine clinical care. Finally, this is a novel demonstration of the value of a large pediatric IBD registry to provide supportive evidence for the regulatory approval of a CD treatment, such as ustekinumab.

## CONFLICT OF INTEREST STATEMENT

Jeremy Adler has received research grants/funding from Johnson & Johnson. Shehzad A. Saeed serves on a speaker's bureau and advisory board for AbbVie. Richard S. Strauss, Anna Sheahan, Renping Zhang, Kim Hung Lo, Lilianne Kim, and Sheri Volger are employees of Johnson & Johnson and own stock/stock options. Richard B. Colletti is a consultant for Johnson & Johnson. The remaining authors declare no conflict of interest.

## Supporting information

Table S1. Ustekinumab exposure.

Table S2. Change from baseline to Week 52 in growth parameters.

Table S3. Change from baseline to Week 52 in laboratory parameters.

Table S4. Discontinuation of ustekinumab through Week 52.

Figure S1. Intention‐to‐treat analysis of clinical effectiveness endpoints at Week 52 in [a] all patients with CD treated with ustekinumab, and [b] patients with moderately‐to‐severely active CD treated with ustekinumab. CD, Crohn's disease; CI, confidence interval; ICE, intercurrent event; M/S, moderately‐to‐severely active CD (sPCDAI ≥30 at baseline); sPCDAI, short Pediatric Crohn's disease Activity Index. Note: For this intention‐to‐treat analysis, patients with an ICE and those with missing data at Week 52 were considered not have achieved clinical remission. The Week‐52 window was defined as Week 52 ± 16 weeks. Week 52 was calculated as the date of the first dose of ustekinumab plus 365 days. The 95% CI was estimated based on the Wilson method. ^a^Clinical remission was defined as sPCDAI ≤10 at Week 52; ^b^Corticosteroid‐free clinical remission was defined as sPCDAI ≤10 without use of corticosteroids at Week 52.

Figure S2. Clinical remission at Week 52 among all patients with CD treated with ustekinumab who increased ustekinumab dose or frequency. CD, Crohn's disease; CI, confidence interval; ICE, intercurrent event; sPCDAI, short Pediatric Crohn's disease Activity Index. Note: For this observed case analysis, patients with missing clinical remission status at Week 52 after accounting for ICEs were excluded. The Week‐52 window was defined as Week 52 ± 16 weeks. Week 52 was calculated as the date of the first dose of ustekinumab plus 365 days. The 95% CI was estimated based on the Wilson method. ^a^Clinical remission was defined as sPCDAI ≤10 at Week 52.

## Data Availability

The data sharing policy of Johnson & Johnson is available at https://innovativemedicine.jnj.com/our-innovation/clinical-trials/transparency. As noted on this site, requests for access to the study data can be submitted through Yale Open Data Access [YODA] Project site at http://yoda.yale.edu.
