# Supplementary material for: Effectiveness and safety of ustekinumab in pediatric Crohn's disease: Results of the REALITI study
Source: J Pediatr Gastroenterol Nutr. 2026 Mar 2;82(5):1242–50. doi: 10.1002/jpn3.70372 (PMC13150951; doi:10.1002/jpn3.70372)
Supplement: Supplementary file 1 — Table S1. Ustekinumab exposure. [file JPN3-82-1242-s006.docx]

| **n (%)** | **Pediatric Patients** | **Young Adult Patients** |
| --- | --- | --- |
| Initial induction dose, N | 334 | 118 |
| 0.5 to <1.65 mg/kg/week (equivalent to 260 to 520 mg IV)^a^ | 309 (92.5) | 110 (93.2) |
| Initial maintenance dose, N | 318 | 120 |
| 90 mg every 12 weeks | 3 (0.9) | 0 |
| 90 mg every 8 weeks | 264 (83.0) | 106 (88.3) |
| 90 mg every 6 weeks | 7 (2.2) | 4 (3.3) |
| 90 mg every 4 weeks | 23 (7.2) | 5 (4.2) |
| Other | 21 (6.6) | 5 (4.2) |
| Final maintenance dose, N | 314 | 119 |
| 90 mg every 12 weeks | 1 (0.3) | 0 |
| 90 mg every 8 weeks | 164 (52.2) | 77 (64.7) |
| 90 mg every 6 weeks | 23 (7.3) | 12 (10.1) |
| 90 mg every 4 weeks | 108 (34.4) | 26 (21.8) |
| Other | 18 (5.7) | 4 (3.4) |

**Table S1.** Ustekinumab exposure.

Abbreviation: IV, intravenous.

^a^ Induction dose was calculated as the sum of all documented ustekinumab doses from ustekinumab initiation up to 8 weeks after the date of the first ustekinumab dose. If a patient received subcutaneous dosing during this interval, the dose was adjusted by 70% to account for the bioavailability of ustekinumab.
