# Supplementary material for: Effectiveness and safety of ustekinumab in pediatric Crohn's disease: Results of the REALITI study
Source: J Pediatr Gastroenterol Nutr. 2026 Mar 2;82(5):1242–50. doi: 10.1002/jpn3.70372 (PMC13150951; doi:10.1002/jpn3.70372)
Supplement: Supplementary file 2 — Table S2. Change from baseline to Week 52 in growth parameters. [file JPN3-82-1242-s005.docx]

| **z-score**  **Median (IQR)** | **Pediatric Patients**  **(N=348)** | | | **Young Adult Patients**  **(N=131)** | | |
| --- | --- | --- | --- | --- | --- | --- |
|  | **Baseline^a^** | **Week 52^b^** | **Change** | **Baseline^a^** | **Week 52^b^** | **Change** |
| Weight | -0.30 (-1.34; 0.49)  n=290 | -0.11 (-1.14; 0.63)  n=284 | 0.13 (-0.16; 0.51)  n=238 | 0.09 (-0.97; 0.86)  n=83 | 0.19 (-0.53; 0.78)  n=43 | 0.21 (-0.18; 0.48)  n=37 |
| Height | -0.43 (-1.17; 0.36)  n=289 | -0.47 (-1.30; 0.30)  n=284 | -0.01 (-0.15; 0.14)  n=238 | -0.33 (-0.88; 0.45)  n=83 | -0.09 (-0.85; 0.47)  n=43 | -0.04 (-0.11; 0.08)  n=37 |
| BMI | -0.25 (-1.26; 0.68)  n=289 | -0.08 (-0.80; 0.84)  n=284 | 0.14 (-0.21; 0.59)  n=238 | -0.21 (-0.83; 0.98)  n=83 | -0.07 (-0.80; 0.82)  n=43 | 0.32 (-0.18; 0.52)  n=37 |

**Table S2.** Change from baseline to Week 52 in growth parameters.

Abbreviations: BMI, body mass index; IQR, interquartile range.

^a^ Baseline value is defined as the non-missing measurement closest to the index date within the baseline window (i.e., from ‑12 weeks to +2 weeks from the index date); the index date is defined as the date of the first dose of ustekinumab.

^b^ The Week 52 value for the endpoint is defined as the non-missing measurement closest to Week 52 within the Week 52 window. The Week 52 window was defined as Week 52 ± 16 weeks. Week 52 is calculated as the date of the first dose of ustekinumab plus 365 days.
