# Supplementary material for: Effectiveness and safety of ustekinumab in pediatric Crohn's disease: Results of the REALITI study
Source: J Pediatr Gastroenterol Nutr. 2026 Mar 2;82(5):1242–50. doi: 10.1002/jpn3.70372 (PMC13150951; doi:10.1002/jpn3.70372)
Supplement: Supplementary file 3 — Table S3. Change from baseline to Week 52 in laboratory parameters. [file JPN3-82-1242-s002.docx]

| **Median (IQR)** | **Pediatric Patients**  **(N=348)** | | | **Young Adult Patients**  **(N=131)** | | |
| --- | --- | --- | --- | --- | --- | --- |
|  | **Baseline^a^** | **Week 52^b^** | **Change** | **Baseline^a^** | **Week 52^b^** | **Change** |
| Hematocrit (%) | 37.35 (34.90; 39.85)  n=240 | 38.60 (36.00; 41.00)  n=225 | 1.10 (-1.00; 3.50)  n=163 | 37.80 (34.95; 40.90)  n=100 | 39.10 (36.65; 41.95)  n=76 | 1.20 (-1.65; 3.00)  n=60 |
| Albumin (g/L) | 40.0 (35.0; 43.0)  n=235 | 42.0 (38.0; 44.0)  n=219 | 2.0 (-1.0; 6.0)  n=155 | 39.0 (33.0; 43.0)  n=99 | 42.0 (38.0; 45.0)  n=73 | 3.0 (0.0; 7.0)  n=59 |
| ESR (mm/h) | 22.0 (11.0; 37.0)  n=214 | 14.0 (8.0; 25.0)  n=190 | -5.0 (-17.0; 5.5)  n=136 | 18.0 (11.0; 44.0)  n=87 | 14.0 (8.0; 28.5)  n=64 | -4.5 (-20.0; 3.0)  n=50 |

1. **Table S3.** Change from baseline to Week 52 in laboratory parameters.
2. Abbreviations: ESR, erythrocyte sedimentation rate; IQR, interquartile range.

^a^ Baseline value is defined as the non-missing measurement closest to the index date within the baseline window (i.e., from ‑12 weeks to +2 weeks from the index date); the index date is defined as the date of the first dose of ustekinumab.

^b^ The Week 52 value for the endpoint is defined as the non-missing measurement closest to Week 52 within the Week 52 window. The Week 52 window was defined as Week 52 ± 16 weeks. Week 52 is calculated as the date of the first dose of ustekinumab plus 365 days.
