# Supplementary material for: Effectiveness and safety of ustekinumab in pediatric Crohn's disease: Results of the REALITI study
Source: J Pediatr Gastroenterol Nutr. 2026 Mar 2;82(5):1242–50. doi: 10.1002/jpn3.70372 (PMC13150951; doi:10.1002/jpn3.70372)
Supplement: Supplementary file 4 — Table S4. Discontinuation of ustekinumab through Week 52. [file JPN3-82-1242-s003.docx]

|  | **Pediatric Patients**  **N=348** | **Young Adult Patients**  **N=131** |
| --- | --- | --- |
| Patients who discontinued study agent, n (%) | 73 (21.0) | 30 (22.9) |
| Reason for discontinuation |  |  |
| Primary non-responder^a^ | 37 (10.6) | 10 (7.6) |
| Secondary failure (worsening of CD)^b^ | 14 (4.0) | 12 (9.2) |
| Intolerant^c^ | 2 (0.6) | 0 |
| Serious adverse event | 1 (0.3) | 0 |
| Insurance/other financial concern | 3 (0.9) | 0 |
| Other | 11 (3.2) | 5 (3.8) |
| Unknown/not reported | 5 (1.4) | 3 (2.3) |

1. **Table S4.** Discontinuation of ustekinumab through Week 52.
2. Abbreviation: CD, Crohn’s disease.

^a^ Ustekinumab was never effective or the initial response was inadequate.
^b^ Ustekinumab was initially effective, then lost effectiveness.
^c^ Includes acute reactions after administration of medication and delayed administration reactions.
