# Supplementary material for: Effectiveness and safety of ustekinumab in pediatric Crohn's disease: Results of the REALITI study
Source: J Pediatr Gastroenterol Nutr. 2026 Mar 2;82(5):1242–50. doi: 10.1002/jpn3.70372 (PMC13150951; doi:10.1002/jpn3.70372)
Supplement: Supplementary file 5 — Figure S1. Intention‐to‐treat analysis of clinical effectiveness endpoints at Week 52 in [a] all patients with CD treated with ustekinumab, and [b] patients with moderately‐to‐severely active CD treated with ustekinumab. CD, Crohn's disease; CI, confidence interval; ICE, intercurrent event; M/S, moderately‐to‐severely active CD (sPCDAI ≥30 at baseline); sPCDAI, short Pediatric Crohn's disease Activity Index. Note: For this intention‐to‐treat analysis, patients with an ICE and those with missing data at Week 52 were considered not have achieved clinical remission. The Week‐52 window was defined as Week 52 ± 16 weeks. Week 52 was calculated as the date of the first dose of ustekinumab plus 365 days. The 95% CI was estimated based on the Wilson method. aClinical remission was defined as sPCDAI ≤10 at Week 52; bCorticosteroid‐free clinical remission was defined as sPCDAI ≤10 without use of corticosteroids at Week 52. [file JPN3-82-1242-s001.docx]

**
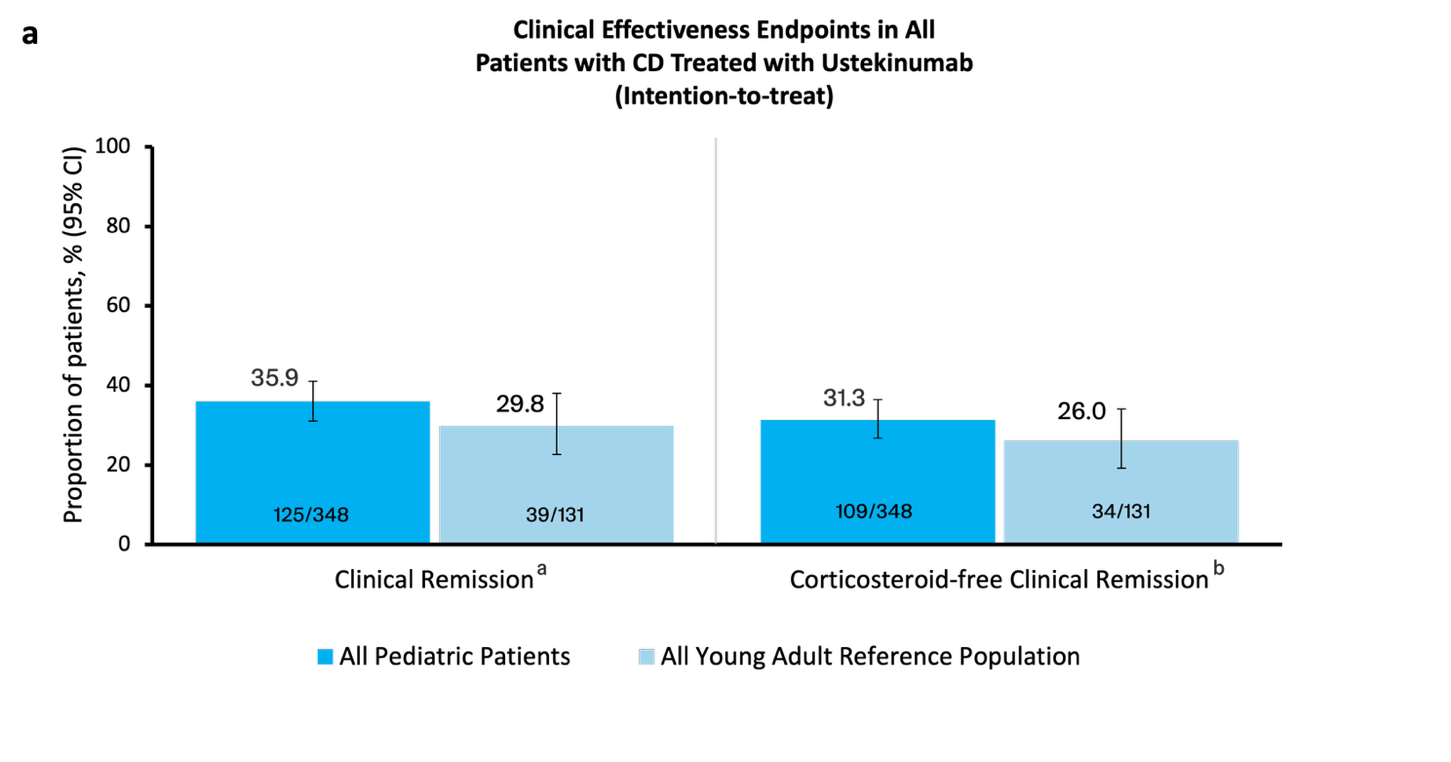
**

**
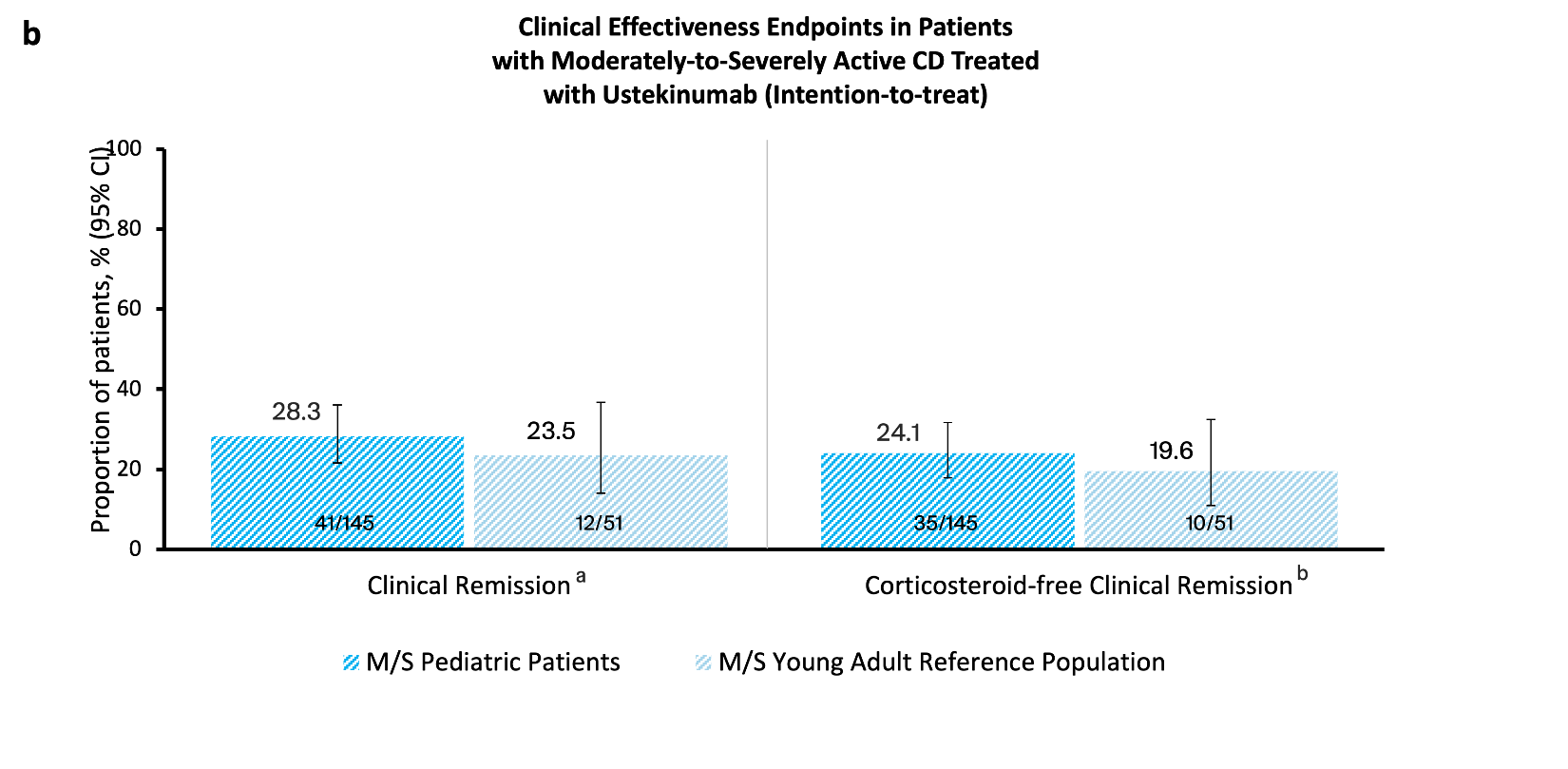
**

**Figure S1.**

Intention-to-treat analysis of clinical effectiveness endpoints at Week 52 in [a] all patients with CD treated with ustekinumab, and [b] patients with moderately-to-severely active CD treated with ustekinumab. CD, Crohn’s disease; CI, confidence interval; ICE, intercurrent event; M/S, moderately-to-severely active CD (sPCDAI ≥30 at baseline); sPCDAI, short Pediatric Crohn’s disease Activity Index. Note: For this intention-to-treat analysis, patients with an ICE and those with missing data at Week 52 were considered not have achieved clinical remission. The Week 52 window was defined as Week 52 ± 16 weeks. Week 52 was calculated as the date of the first dose of ustekinumab plus 365 days. The 95% CI was estimated based on the Wilson method. ^a^Clinical remission was defined as sPCDAI ≤10 at Week 52; ^b^Corticosteroid-free clinical remission was defined as sPCDAI ≤10 without use of corticosteroids at Week 52.
