# Supplementary material for: Effectiveness and safety of ustekinumab in pediatric Crohn's disease: Results of the REALITI study
Source: J Pediatr Gastroenterol Nutr. 2026 Mar 2;82(5):1242–50. doi: 10.1002/jpn3.70372 (PMC13150951; doi:10.1002/jpn3.70372)
Supplement: Supplementary file 6 — Figure S2. Clinical remission at Week 52 among all patients with CD treated with ustekinumab who increased ustekinumab dose or frequency. CD, Crohn's disease; CI, confidence interval; ICE, intercurrent event; sPCDAI, short Pediatric Crohn's disease Activity Index. Note: For this observed case analysis, patients with missing clinical remission status at Week 52 after accounting for ICEs were excluded. The Week‐52 window was defined as Week 52 ± 16 weeks. Week 52 was calculated as the date of the first dose of ustekinumab plus 365 days. The 95% CI was estimated based on the Wilson method. aClinical remission was defined as sPCDAI ≤10 at Week 52. [file JPN3-82-1242-s004.docx]

**
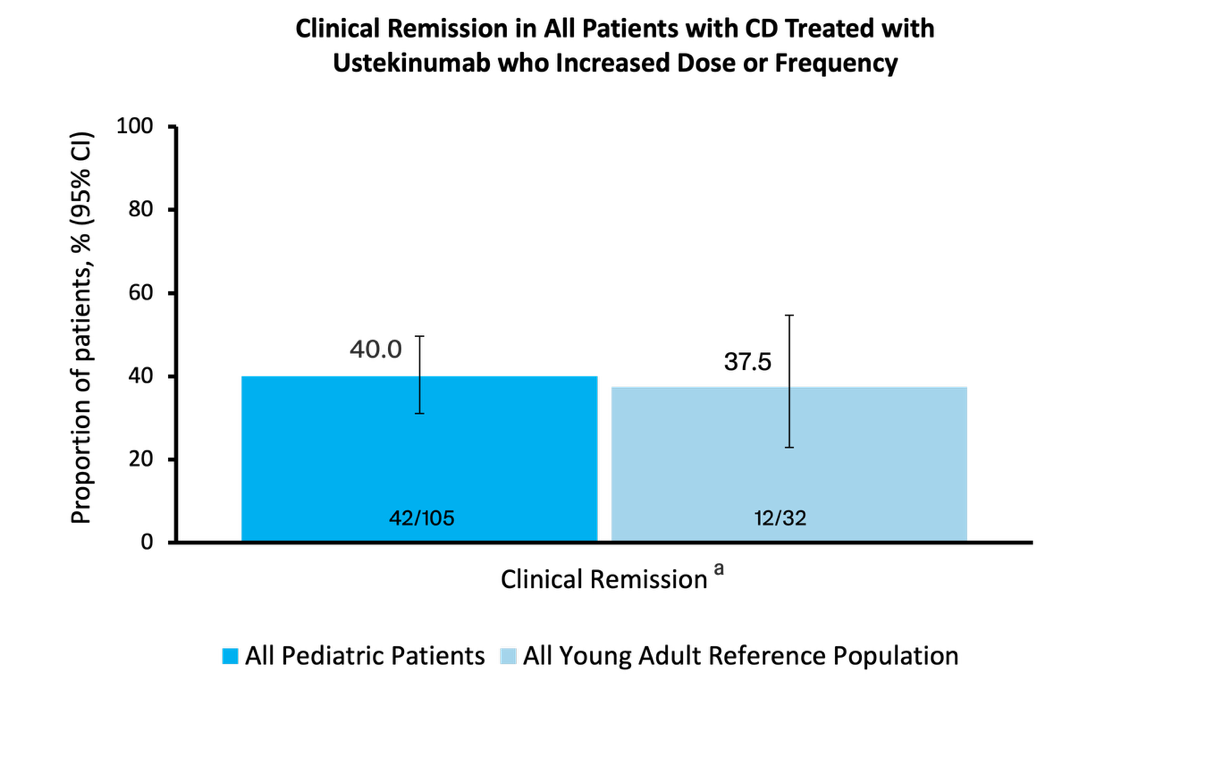
**

**Figure S2.**

Clinical remission at Week 52 among all patients with CD treated with ustekinumab who increased ustekinumab dose or frequency. CD, Crohn’s disease; CI, confidence interval; ICE, intercurrent event; sPCDAI, short Pediatric Crohn’s disease Activity Index. Note: For this observed case analysis, patients with missing clinical remission status at Week 52 after accounting for ICEs were excluded. The Week 52 window was defined as Week 52 ± 16 weeks. Week 52 was calculated as the date of the first dose of ustekinumab plus 365 days. The 95% CI was estimated based on the Wilson method. ^a^Clinical remission was defined as sPCDAI ≤10 at Week 52.
